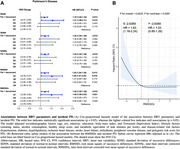# Reduced heart rate variability associated with higher risk of Parkinson's disease: A prospective cohort study

**DOI:** 10.1002/alz70856_105380

**Published:** 2026-01-07

**Authors:** Ruihan Wang, Kai Zhou, Qing Gao, Qin Chen

**Affiliations:** ^1^ West China Hospital of Sichuan University, Chengdu, Sichuan, China; ^2^ University of Electronic Science and Technology of China, Chengdu, Sichuan, China

## Abstract

**Background:**

Cross‐sectional studies have suggested that patients with Parkinson's disease (PD) have significantly lower heart rate variability (HRV) than healthy controls. However, the role of HRV as an early biomarker for PD remains unclear. The objective of this study was to investigate the association between HRV and PD risk and its underlying mechanisms.

**Method:**

In a prospective cohort study based on the UK Biobank, 48,202 participants without PD and dementia at baseline who had available 15‐second resting electrocardiogram (ECG) data (*n* = 48,202) were included. The participants were followed up for an average of 12.24 years and some were diagnosed with PD (*n* = 307). Cox proportional hazards models were used to examine the association between HRV parameters and PD risk, after adjusting for multiple confounders. A nested case‐control study was conducted within the cohort to further investigate temporal trends in HRV. Mediation analysis was used to explore the underlying mechanisms driven by brain structure and peripheral inflammation biomarkers.

**Result:**

Lower HRV parameters were significantly associated with an increased risk of PD. Notably, an L‐shaped association was observed between inter‐beat interval corrected root mean square of successive differences (RMSSDc) and PD risk, where only lower RMSSDc levels (below ‐2.6369) were associated with an increased PD risk (HR = 1.63, 95% CI 1.18–2.24). Temporal trend analysis found that HRV levels of patients with PD started to be lower than those of controls approximately 10 years before diagnosis. Mediation analysis revealed that thalamus‐related fiber tracts and peripheral blood inflammatory markers (monocyte count and four plasma proteins, including histamine N‐methyltransferase, interleukin‐13 receptor subunit alpha‐1, secretogranin‐2, and BAG family molecular chaperone regulator 3) mediated the association between HRV and PD risk.

**Conclusion:**

Our findings provide preliminary evidence supporting an association between reduced HRV and higher PD risk, suggesting that HRV may serve as an early, convenient, and noninvasive biomarker of PD risk up to a decade before diagnosis.